# Jejunogastric intussusception associated with jejunojejunal intussusception (double telescoping) occurring 20 years after gastrojejunostomy

**DOI:** 10.1186/s12245-024-00612-6

**Published:** 2024-03-11

**Authors:** Souhaib Atri, Mahdi Hammami, Amine Sebai, Rany Aifia, Meriem Ben Brahim, Youssef Chaker, Fadhel Samir Fteriche, Montassar Kacem

**Affiliations:** Hopital La Rabta, Tunis, Tunisia

**Keywords:** Gastrojejunostomy, Jejunogastric intussusception, Case report

## Abstract

**Introduction:**

Jejunogastric intussusception (JGI) is a rare but potentially lethal complication following gastrectomy or gastrojejunostomy surgeries. Diagnosis of this condition can be challenging due to its rarity and non-specific symptoms. This article presents a case report of a 60-year-old male with a history of trans mesocolic gastrojejunostomy who developed acute symptoms of JGI.

**Case report:**

The patient presented with acute epigastric pain, vomiting, and hematemesis. Physical examination and laboratory tests indicated dehydration, tachycardia, and leukocytosis. Computed tomography (CT) revealed intussuscepted loops within the stomach. Emergency laparotomy was performed, and the intussusception was manually reduced without the need for resection. The patient recovered well and was discharged five days post-surgery.

**Discussion:**

Retrograde jejunogastric intussusception is a rare complication, often occurring years after gastric surgery. It can be classified into acute and chronic forms, with the former presenting with intense pain and potential hematemesis. The condition can arise in different surgical contexts and even spontaneously. The cause of JGI remains unclear, but factors such as hyperacidity, abnormal motility, and increased intra-abdominal pressure have been implicated. Diagnosis can be made through endoscopy or alternative imaging modalities such as CT. Surgical intervention is the treatment of choice, with various options available based on intraoperative findings.

**Conclusion:**

Retrograde jejunogastric intussusception is challenging to diagnose and treat due to its rarity and lack of understanding of its causes. Imaging techniques and endoscopy play important roles in diagnosis, while surgery remains the primary treatment option. Vigilance is necessary among medical professionals to consider JGI in cases of acute abdominal pain and vomiting following gastric surgery, allowing for prompt diagnosis and intervention to prevent bowel necrosis. Further research is needed to establish optimal surgical strategies and evaluate recurrence rates.

## Background

Jejunogastric intussusception (JGI) is a rare but potentially lethal complication that can occur after gastrectomy or gastrojejunostomy surgeries. This condition involves telescoping the jejunum into the stomach, and if left undiagnosed, the mortality rate of the acute form can reach 50% [1]. Patients with a history of gastric surgery are at a higher risk of developing JGI, but preoperative awareness of this condition can be challenging due to its rarity and non-specific symptoms.

## Case report

A 60 years old male, with a history of trans mesocolic gastrojejunostomy for pyloric stenosis 20 years ago, presented to the emergency department with complaints of acute onset epigastric pain with multiple episodes of vomiting initially bilious and then hematemesis. The patient had a routine endoscopy 24 h prior.

Physical examination showed dehydration and tachycardia. Abdominal examination showed epigastric tenderness. An upper midline abdominal scar was seen with no signs of incisional hernia. An ill-defined mass was palpable in the epigastric region. It was mobile from side to side and with respiration.

Biology revealed leukocytosis and elevated CRP. Hemoglobin was normal. The rest of the parameters were within normal limits.

**Fig. 1:**
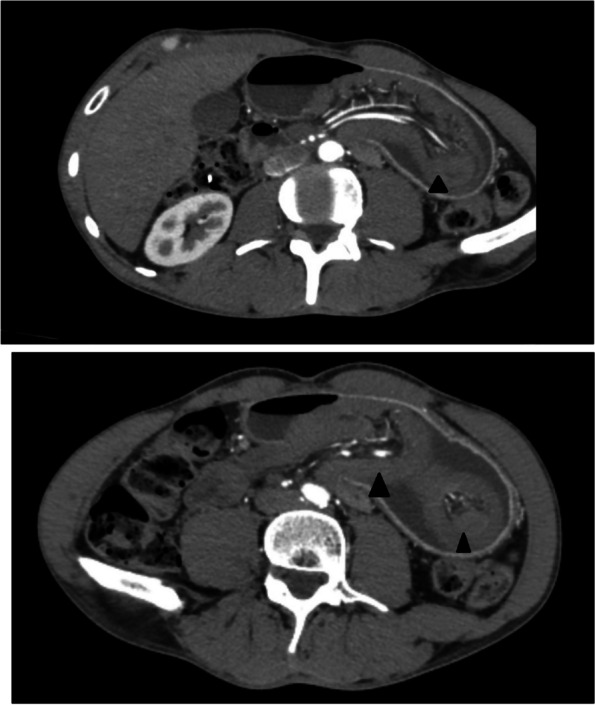
Enhanced CT imaging showing jejunal loops inside the stomach (white arrows)

After conducting a computed tomography (CT), a significantly enlarged stomach filled with fluid was observed, and the classical swirling appearance of intussuscepted loops was visible within the stomach's body (Fig. 1). However, no anomalies related to the intestinal wall's enhancement were detected.

**Fig. 2:**
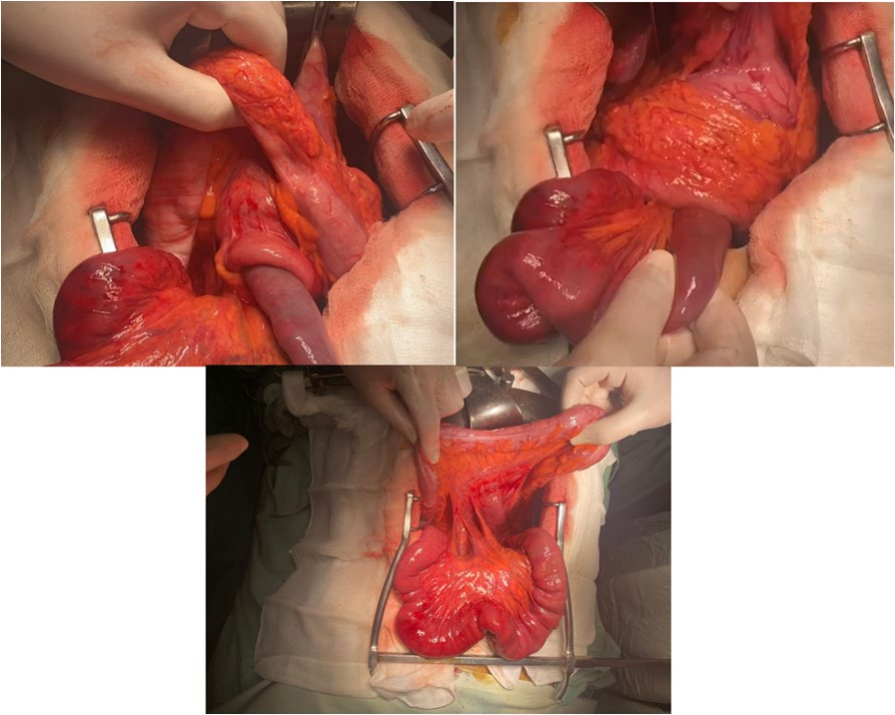
Retrograde jejunogastric intussusception of the efferent limb at the trans mesocolic gastrojejunostomy site

Following the first stage of resuscitation, an emergency laparotomy was performed. During the operation, it was discovered that there was a retrograde JGI at the previous retro colic gastrojejunostomy site (Fig. 2). With careful manipulation, the intussuscepted loop was manually reduced, revealing another segment of the jejunum that was telescoping within the loop. Fortunately, the vitality of the affected intestinal loop was preserved, so there was no need for any resection. The patient's recovery was smooth, and they were discharged from the hospital five days after the surgery. The patient was doing well at six months follow-up.

## Discussion

Retrograde jejunogastric intussusception is a rare acute abdominal condition where small bowel loops get incarcerated and strangulated inside the stomach. It is a rare complication after gastric surgery, with less than 0.1% incidence [1] and around 200 cases reported in the world literature since the first case of jejunogastric intussusception after gastrojejunostomy was reported by Bozzi in 1914 [2].

The duration between the gastric surgery and the occurrence of the JGI varies from a few days to few decades [3]. Vaidya et al. [3] described a JGI occurring 40 years after Billroth II gastrectomy and Tokue et al. [4] reported a JGI occurring 55 years after gastrojejunostomy. In our case, the JGI occurred after 20 years.

There are two distinct forms of JGI: acute and chronic [1]. The acute form is characterized by the incarceration and strangulation of the intussuscepted loop, resulting in intense colicky epigastric pain, vomiting, and potential hematemesis. In contrast, the chronic form is characterized by spontaneous reduction of intussusception. In the case at hand, the patient exhibits symptoms consistent with the acute form, including acute severe epigastric pain, vomiting, and subsequent hematemesis.

**Fig. 3:**
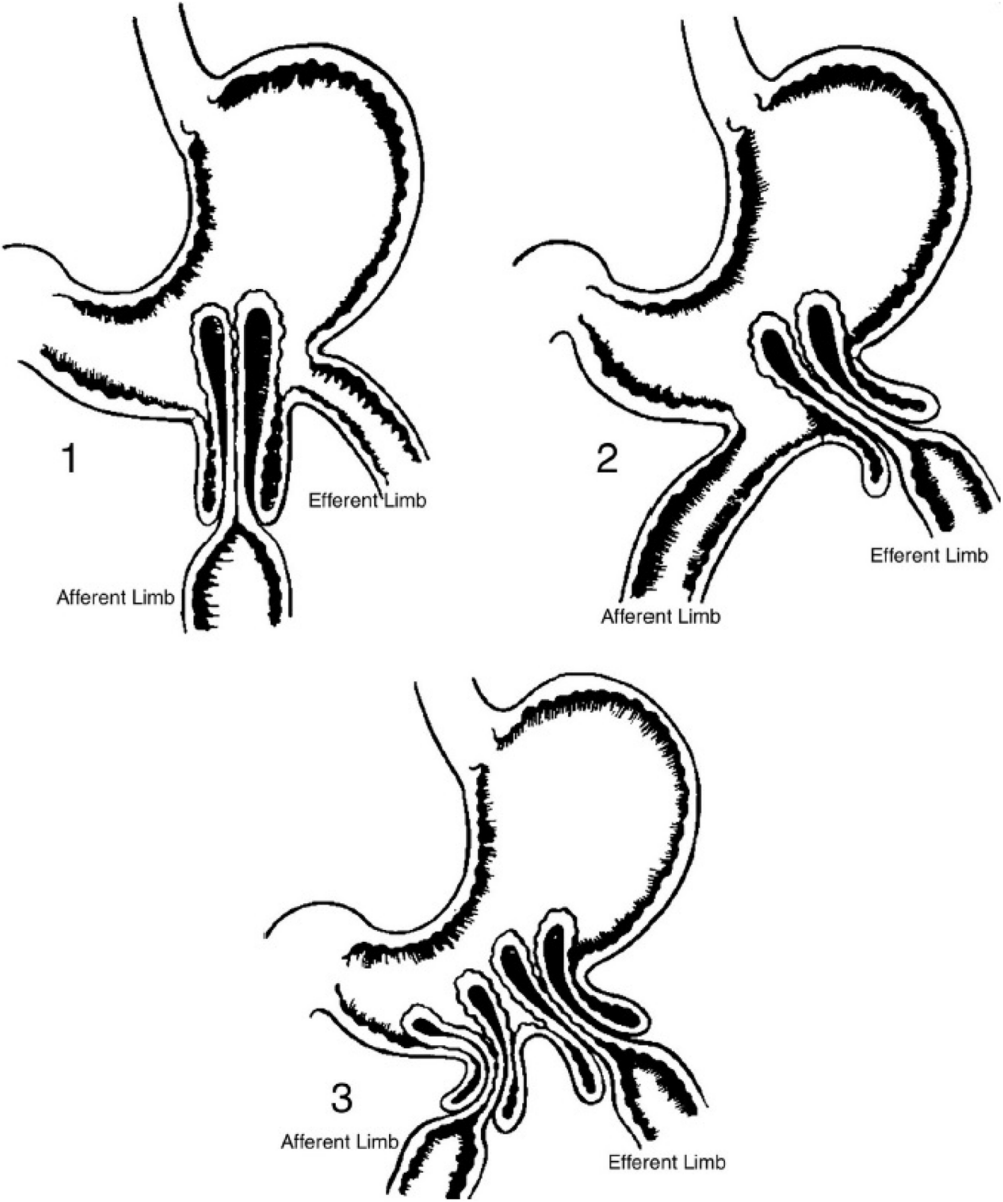
Types of Jejunogastric Intussusception. 1 Afferent limb intussusception. 2 Efferent limb intussusception. 3 Intussusception of both limbs

The condition is classified into three anatomical types: type I (afferent loop intussusception), type II (retrograde efferent loop intussusception), and type III (a combination of type I and type II) (Fig. 3) [5]. Type II or retrograde efferent loop intussusception is the most common, accounting for 80% of cases [6]. As it is pointed in our case, our patient presented type II JGI.

The occurrence of JGI is not limited to gastrojejunostomy, as it can also complicate other gastric surgeries such as Billroth II gastrectomy, distal gastrectomy with Roux-en-Y reconstruction [7], and Whipple procedure [8]. Interestingly, there have been documented cases of JGI that develop without any prior history of gastric surgery, as reported by Caruso et al. [9] in 2001. Therefore, while our patient experienced JGI following a gastrojejunostomy, it is crucial to recognize that this condition can arise in various surgical contexts or even spontaneously.

The cause of JGI has sparked debate, with various theories proposed. Some suggest that functional factors like hyperacidity or spasms lead to increased peristalsis. Others point to mechanical factors such as adhesions, heightened intra-abdominal pressure, post-vagotomy dilation, and stomach atony with retrograde peristalsis. However, the precise mechanism remains elusive. Retrograde peristalsis, a common occurrence in the small bowel, is often cited as a contributing factor to JGI. Antiperistaltism may be influenced by the segmented motor activity of the small bowel and hyperacidity, which can occur following limited antral resections or gastroenteroanastomosis.

Endoscopy is considered the primary diagnostic approach for evaluating retrograde jejunogastric intussusception (JGI) due to its ability to directly assess the condition of the intussuscepted bowel and detect signs of ischemia or strangulation [10]. However, in cases where endoscopy is unavailable, alternative imaging modalities can provide valuable diagnostic clues. In our specific case, a computed tomography (CT) scan was performed, revealing a significantly enlarged stomach filled with fluid. Within the gastric body, characteristic swirling appearances of intussuscepted loops were observed, indicating the presence of JGI. Notably, the CT scan did not reveal any abnormalities in the enhancement of the intestinal wall. While endoscopy was not performed in our case, it remains a crucial tool for the initial diagnosis of JGI, allowing for direct visualization and assessment of the condition of the intussuscepted bowel.

Surgery remains the treatment of choice for retrograde jejunogastric intussusception (JGI), with a range of surgical procedures available based on intraoperative findings. Options may include manual reduction of the intussuscepted loops with fixation of the jejunal limb, which was the case for our patient, resection and revision of the anastomosis, or creation of a Roux-en-Y bypass, depending on the viability of the bowel. Recurrence rates between different surgical approaches are poorly documented due to the rarity of this complication. The mortality rate is also poorly documented, but it can atteign 10% [11]. Therefore, individualized operative management should be determined based on intraoperative findings and the patient's overall health status. Future research is needed to compare different operative strategies and evaluate their impact on recurrence rates, with the aim of establishing optimal interventions for JGI.

## Conclusion

The rarity of retrograde jejunogastric intussusception and the lack of understanding of its causes make it difficult to diagnose and treat. Imaging techniques such as ultrasonography and CT scans are useful in identifying the condition, while upper endoscopy can be both diagnostic and therapeutic. Surgical management is the primary treatment option, which may include intestinal resection, anastomosis revision, or jejunum fixation to prevent recurrence. With prompt diagnosis and immediate surgical intervention, it may be possible to prevent necrosis in the intussuscepted bowel. However, given the condition's complexity, medical professionals must remain vigilant and consider the possibility of retrograde jejunogastric intussusception when presented with cases of acute abdominal pain and vomiting after gastric surgery.

## Data Availability

Not applicable.
